# Chronic recurrent multifocal osteomyelitis: a case report

**DOI:** 10.1186/s13052-018-0463-3

**Published:** 2018-02-17

**Authors:** Maria Francesca Gicchino, Mario Diplomatico, Carmela Granato, Daniela Capalbo, Pierluigi Marzuillo, Alma Nunzia Olivieri, Emanuele Miraglia del Giudice

**Affiliations:** Department of Woman and Child and General and Specialized Surgery, University of the Study of Campania “Luigi Vanvitelli”, Via Luigi de Crecchio 4, 80138 Naples, Italy

**Keywords:** Chronic recurrent multifocal osteomyelitis, CRMO, Bone pain

## Abstract

**Background:**

Chronic recurrent multifocal osteomyelitis (CRMO), also known as chronic nonbacterial osteomyelitis, is a rare, noninfectious inflammatory disorder that causes multifocal bone lesions with swelling and pain. Lytic and sclerotic bone lesions could be found on X-ray. Short tau inversion recovery magnetic resonance imaging (STIR MRI) shows bone marrow oedema, bone expansion, lytic areas and periosteal reaction. CRMO is characterized by periodic exacerbations and remissions of unclear/unknown pathogenesis.

**Case presentation:**

A 10 years old girl, suffering from pain in her right shoulder since the age of 9 years presented to our Department. Thanks to clinical data, laboratoristic and radiological findings and bone biopsy CRMO was diagnosed. So patient started anti-inflammatory treatment and her conditions improved.

**Conclusions:**

In a child with bone pain should be considered also rare condition as CRMO to perform a correct diagnosis and start an adequate treatment avoiding complications such as bone damage. This condition should be suspected in a child with recurrent bone pain, modest increase of inflammatory indices, lytic or sclerotic bone lesion on X Ray. Typical CRMO localizations are metaphyses of long bones, pelvis, clavicle, vertebral column, sternum, ribs, jaw, but any bone can be involved. The most common CRMO differential diagnosis is represented by infections, malignant bone tumors, Langerhans Cells Histiocytosis (LCH).

## Background

Chronic recurrent multifocal osteomyelitis (CRMO), also known as chronic nonbacterial osteomyelitis, is a rare, noninfectious inflammatory disorder that causes multifocal lytic bone lesions characterized by periodic exacerbations and remissions [1, 2]. This condition affects children and adolescents with a female: male ratio of 4:1. CRMO is still considered a rare disease with a prevalence of 1–2/10^6^. CRMO prevalence is probably underestimated [3]. This condition was described by Giedion for the first time in1972 [4]. The differential diagnosis includes infectious osteomyelitis, malignancy (osteosarcoma, Ewing’s sarcoma, leukemia, Non-Hodgkin lymphoma), benign bone lesion (as osteoid osteoma), Langerhans cells histiocytosis (LCH). The diagnosis is of exclusion, based on the clinical and radiological data, in fact blood tests show a modest elevation of inflammations parameters and leukocytosis in the majority of cases. Biopsy is needed to exclude infectious osteomyelitis or malignant bone tumor [5–7].CRMO is characterized by bone pain with insidious onset. Skeletal manifestations are unifocal or multifocal, any bone can be envolved. Typical localizations are metaphyses of the long bones (74%), pelvis (38%), vertebral column (46%), clavicle (25%), jaw (18%), sternum (8%), ribs (8%) [8]. The involvement of clavicle, sternum or jaw suggests a CRMO diagnosis [9, 10]. The overhead skin can be erythematous and swollen. Arthritis of adjacent and distal joints could manifest up to 80% of patients [11]. CRMO could be associated with peripheral arthritis, sacroileitis, inflammatory bowel diseases (in particular with Crohn’s disease), psoriasis, pyoderma gangrenosum, Sweet syndrome, Wegener’s granulomatosis, Takayatsu’s arteritis [12–15]. Some authors consider CRMO the pediatric equivalent of SAPHO syndrome (Synovitis, Acne, Pustulosis, Hyperostosis, Osteitis), characterized by association of osteoarticular and skin disorders [16].

We could find only a slight leukocytosis and a modest increase of inflammation parameters on blood tests [17]. CRMO pathogenesis is still unclear. It has been suggested that the imbalance between pro-inflammatory cytokines (IL-6, IL-1, TNF α) and anti-inflammatory cytokine (IL-10) could be responsible of CRMO pathogenesis, because these cytokines are involved in bone reabsorption and remodeling through the activation of osteoblasts and osteoclasts [17–22]. Peripheral bloods mononuclear cells from patients with CRMO stimulated in vitro with lipopolisysaccharide (LPS) compared with healthy control cells showed an important increase in IL-1 release [23]. Data from mice with chronic multifocal osteomyelitis and humans with Majeed syndrome (CRMO with dyserythropoietic anemia) suggest that CRMO could belong to the family of autoinflammatory disorders, a group of different conditions characterized by attacks of inflammation that are unprovoked (or triggered by a minor event) and primarily are related to dysregulation of the innate immune system. Many of these syndromes are monogenically inherited. Unlike autoimmune diseases, there is a relative deficiency of both autoantibodies and autoreactive T lymphocytes. The inflammatory response is usually mediated by proinflammatory cytokines, especially Interleukin 1 secreted by granulocytes and monocytes [24]. Mutations in LPIN2, Pstpip2, IL1RN, and FBLIM1 have been found in patients suffered from CRMO and murine models of CRMO [25].

We describe a case of a 10 years old girl presenting with pain in her right shoulder and having the final diagnosis of CRMO with the aim to give to the general pediatrics the key elements to early suspect CRMO and avoid misdiagnoses or late diagnoses.

## Case presentation

**Fig. 1:**
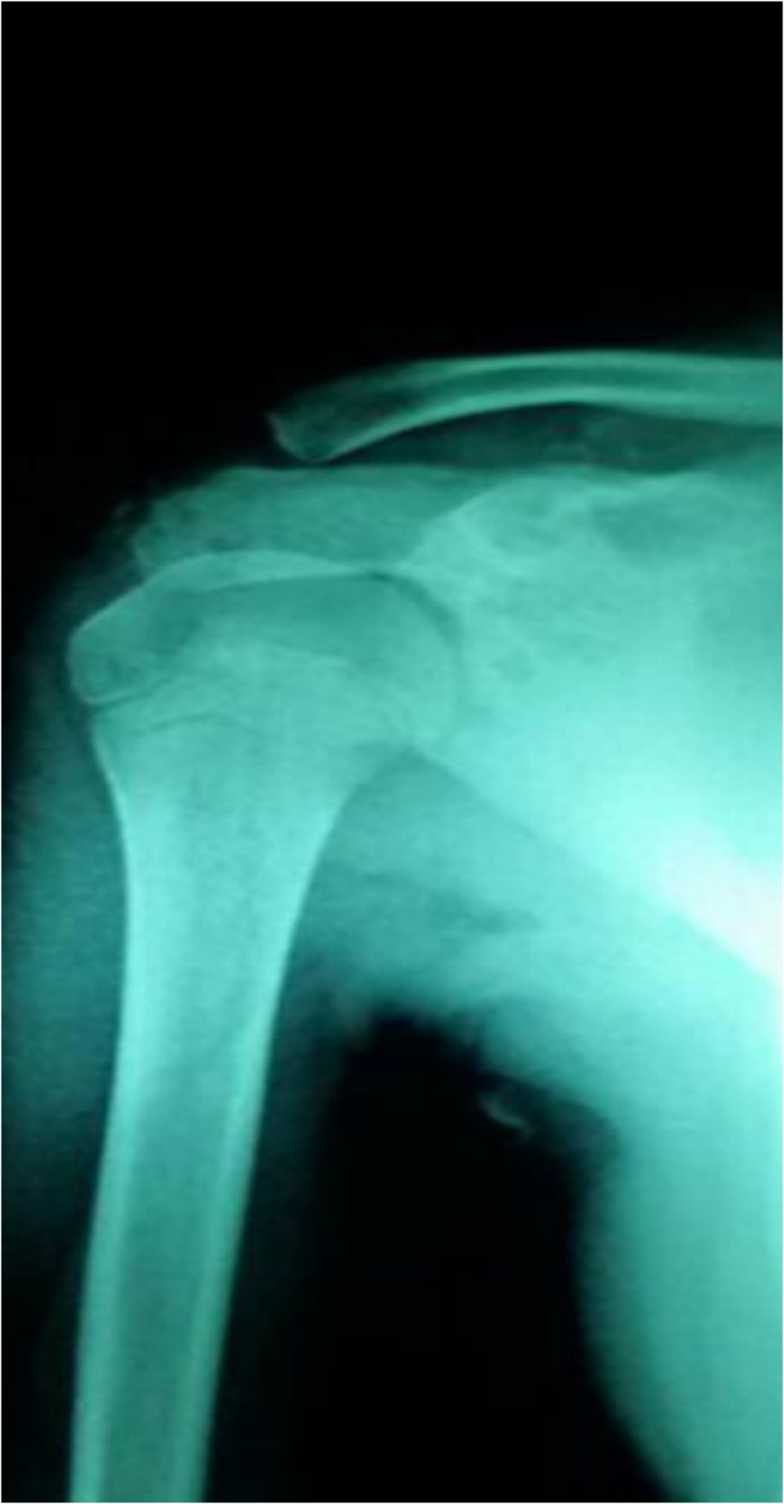
Osteolytic lesion on right shoulder

**Fig. 2:**
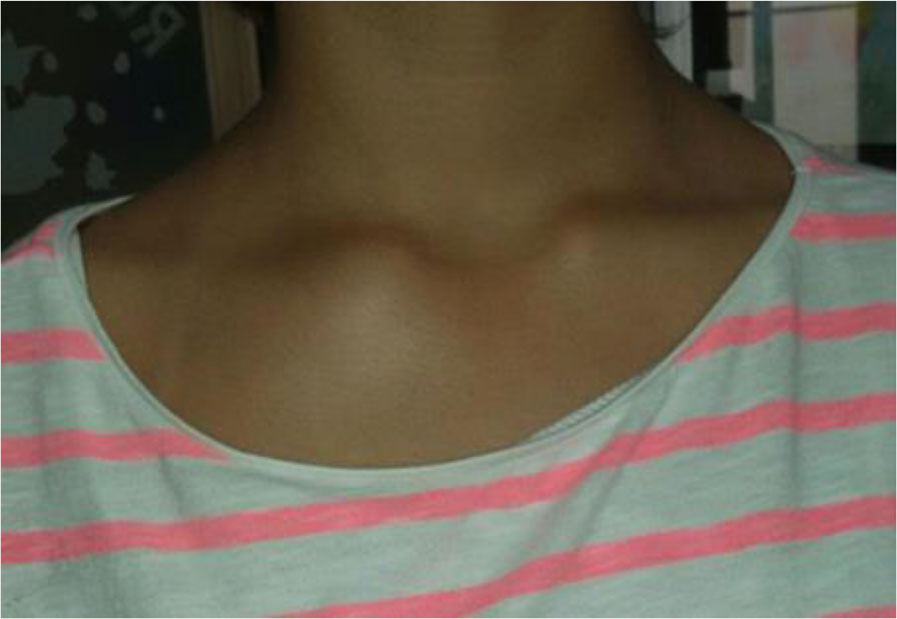
Painful swelling of the sternal end of the right clavicle

**Fig. 3:**
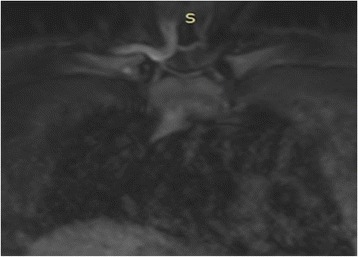
Osteolytic lesion hyperintense on MRI T2 and STIR images

A 10 years old girl, suffering from pain in her right shoulder since the age of 9 years, presented to our Department. She did not recall a precipitating event or a trauma, reported no fever or weakness. There was no relevant personal or family history. Because to persistence of symptoms before coming to our observation an X-ray of her right shoulder revealing an osteolytic lesion was performed (Fig. 1). In order to exclude an infectious osteomyelitis or a malignant tumor, the patient also underwent to a PET-CT showing the presence of a pathological high-uptake of the lesion. Patient’s right shoulder biopsy showed bone infiltration by lymphocytes and neutrophils. No neoplastic cells were identified. All cultures were negative. LCH was excluded since immunohistochemical evaluation of bone marrow for CD1a and S100 expression was negative. This histopathological pattern suggested an inflammatory process, so she assumed steroidal treatment for a week with improvement of symptomatology, but when patient suspended her therapy pain came back again. When she was admitted to our Department she had pain in her right arm and shoulder. Laboratory results showed a discrete increase of erythrocyte sedimentation rate (ESR 34 mm/h, normal value < 20 mm/h) and C-reactive protein (CRP 0,88 mg/dL, normal value < 0,5 mg/dL) with normal complete blood count, liver and renal function as such as abdominal ultrasound and chest X-ray. At MRI, the lesion of the shoulder was hypointense in T1 and hyperintense in T2 and STIR, suggesting an inflammatory process. Suspecting a CRMO we prescribed anti-inflammatory treatment with naproxen. The symptomatology disappeared and the inflammation parameters returned to a normal range 2 months later. Anti-inflammatory treatment was suspended after 3 months of therapy. Five months later, the patient came back to our Department because of pain and swelling in her right clavicle (Fig. 2). The physical examination showed a painful swelling of the sternal end of the right clavicle. Laboratory results indicated a modest increase of inflammation parameters: ESR 29 mm/h (normal value < 20 mm/h), CRP 0.98 mg/dL (normal value < 0.5 mg/dL). Clavicle X-ray showed an osteolytic lesion, ultrasound of sternoclavicular joint revealed articular effusion. On MRI this lesion was hyperintense in T2 and STIR (Fig. 3), so anti-inflammatory treatment with naproxen was started again and the symptoms disappeared after 2 months. Clinical history, physical examination, histopathological pattern were highly suggestive of CRMO. Six months later a STIR MRI to evaluate patient’s bone lesions was performed. The MRI did not show new lesions and previous bone lesions disappeared, so anti-inflammatory treatment has been suspended.

## Discussion

CRMO diagnosis is based on clinical, laboratory and radiologic findings. Laboratory tests are not specific, an increase of inflammatory index can be found, sometimes in association with leukocytosis [9, 10]. The first radiological approach in a child with bone pain is a conventional X-ray that may be normal in the early stage of disease. The first radiological findings are modifications of bone metaphyses close to growth plates, while osteolytic and sclerotic lesions usually appear in the late stages of the disease [21]. STIR MRI is very useful to identify bone lesions and tissue oedema and it is more accurate than bone scintigraphy. CRMO inflammatory lesions appear hypointense in T1-weighted and hyperintense in T2-weighted images [22]. How effective biopsy could be is a still debated topic, in fact histologic features are not specific but it is very important to exclude any other causes of bone pain such as infectious osteomyelitis, a malignant bone tumor or a LCH. Some authors suggest that biopsy could be avoided if a child has classical radiological findings of CRMO or comorbidities, such as Crohn [10, 26, 27]. Some authors have suggested both diagnostic criteria and clinical score to facilitate CRMO diagnosis and to reduce the numbers of bone biopsies (Table 1) [8, 9, 23].

**Table 1:** Diagnostic criteria and diagnostic score for CRMO (modified by references n° 8, 16, 26)

Diagnostic criteria of CRMO proposed by Jansson et al.		Diagnostic criteria proposed by Roderick et al.
**Major criteria:** 1 Radiologically proven osteolytic/−sclerotic bone lesion 2 Multifocal bone lesions 3 PPP or psoriasis 4. Sterile bone biopsy with signs of inflammation and/or fibrosis, sclerosis	**Minor criteria:** 1 Normal blood count and good general state of health 2 CRP and ESR mildly-to-moderately elevated 3 Observation time longer than 6 months 4 Hyperostosis 5 Associated with other autoimmune diseases apart from PPP or psoriasis 6 Grade I or II relatives with autoimmune or autoinflammatory disease, or with CRMO	1 The presence of typical clinical findings: Bone pain +/−localized swelling without significant local or systemic features of inflammation or infection **+** 2 The presence of typical radiological findings: X-ray showing combination of lytic areas, sclerosis and new bone formation, or STIR MRI showing bone marrow oedema +/− bone expansion, lytic areas and periosteal reaction **Associated with** 1 More than one bone (or clavicle alone) without significantly raised CRP (CRP < 30 g/L) or 2 Unifocal disease (other than clavicle), or CRP > 30 g/L, with bone biopsy showing inflammatory changes (plasma cells, osteoclasts, fibrosis or sclerosis) with no bacterial growth whilst not on antibiotic therapy
CRMO is confirmed by two major criteria or one major and three minor criteria		
Diagnostic score proposed by Jansson et al.		
Normal blood cell count	13	Score from 0 to 28 probably not CRMO; score from 29 to 38 uncertain diagnosis, and score values of 39 probably CRMO.
Symmetric lesions	10	
Lesions with marginal sclerosis	10	
Normal body temperature	9	
Vertebral, clavicular, sternal lesions	8	
Radiologically proven lesions	7	
CRP 1 mg/dl	6	
Total clinical score	63	

We presented the case of a girl with a 1 year shoulder pain. Shoulder involvement is not very frequent in CRMO, so bone lesion biopsy was very important in the differential diagnosis. The development of a second lesion at the medial portion of right clavicle (considered a typical site of CRMO) together with previously performed investigation confirmed CRMO diagnosis [8, 9, 23]. To treat CRMO do not exist guidelines, so the treatment is still empiric. Non- Steroidal Anti-Inflammatory drugs (NSAIDs) are the first choice for CRMO treatment not only to keep pain under control but also to prevent bone damage [1]. Oral corticosteroids are used in patients with CRMO that does not respond to NSAIDs [28]. Methotrexate is a well-known treatment in rheumatologic conditions, it represents a second line treatment in CRMO, but further studies are needed [29]. Sulfasalazina is usually used in patients with associated inflammatory bowel disease [30]. Bisphosphonates are indicated in patients with multifocal or spinal involvement [31]. TNF-alfa inhibitors are indicated in patients who do not respond to previous treatments [32]. Also anti-Interleukin1 beta could be a treatment option, but further studies are needed [33]. Our patient is in treatment with anti-inflammatory drugs, and she is well responding to them. The last STIR MRI did not show new lesions and the previous ones disappeared.

## Conclusion

CRMO has an insidious onset of symptoms, with an average diagnosis delay up to 12 months as per some reports. In a child with recurrent bone pain, modest increase of inflammatory indices, lytic or sclerotic lesion on X-ray, bone marrow oedema on STIR MRI, CRMO should always be suspected. Even if typical CRMO localizations are metaphyses of long bones, pelvis, clavicle, vertebral column, sternum, ribs, and jaw, it is important to remember that any bone can be involved to avoid diagnostic delay and to prescribe an adequate treatment. In a child with bone pain also rare condition as CRMO should be considered to perform a correct diagnosis and start an adequate treatment to prevent complications such as bone damage.

## Abbreviations

CRMO: Chronic recurrent multifocal osteomyelitis
CRP: C-reactive protein
DMARDs: Disease-modifying antirheumatic drugs
ERS: Erythrocyte rate sedimentation
IBD: Inflammatory bowel disease
LCH: Langerhans cells histiocytosis
LPS: Lipopolisysaccharide
MRI: Magnetic resonance imaging
NSAIDs: Non steroid anti-inflammatory drugs
SAPHO: Synovitis, Acne, Pustulosis, Hyperostosis, Osteitis syndrome
STIR: Short tau inversion recovery
TC: Computerized Tomography scan

## References

[1] Hedrich CM (2013). Autoinflammatory bone disorders with special focus on chronic recurrent multifocal osteomyelitis (CRMO). Pediatr Rheumatol.

[2] Falip C, Alison M, Bountry N (2013). Chronic recurrent multifocal osteomyelitis (CRMO): a longitudinal case series review. Pediatric Radiol.

[3] Schnabel A, Range U, Hahn G (2016). Unexpectedly high incidences of chronic non-bacterial as compared to bacterial osteomyelitis in children. Rheumatol Int.

[4] Giedion A, Holthusen W, Masel LF (1972). Subacute and chronic “Symmetrical” osteomyelitis. Ann Radiol.

[5] Jibri Z, Sah M (2012). Chronic recurrent multifocal osteomyelic mimicking osteomaosteoide. JBR-BTR.

[6] Borzutzky A, Stern S, Reiff A (2012). Pediatric chronic nonbacterial osteomyelitis. Pediatrics.

[7] Girschick HJ, Monret E, Beer M (2007). Chronic multifocal non bacterial osteomyelitis in hypophosphatasia mimicking malignancy. BMC Pediatr.

[8] Roderick MR, Shah R, Rogers V, Finn A, Ramanan AV (2016). Chronic recurrent multifocal osteomyelitis (CRMO) - advancing the diagnosis. Pediatr Rheumatol Online J..

[9] Jansson AF, Müller TH, Gliera L (2009). Clinical score for nonbacterial osteitis in children and adults. Arthritis Rheum.

[10] Petty RE (2016). Textbook of pediatric rheumatlogy. 7^th^ end.

[11] Girschick HJ, Raab P, Surbaum S (2005). Chronic multifocal non bacterial osteomyelitis in children. Ann Rheum Dis.

[12] Omidi CJ, Siegfried EC (1998). Chronic recurrent multifocal osteomyelitis preceding pyodermagangrenosum and occult ulcerative colitis in a pediatric patient. Pediatr Dermatol.

[13] Dagan O, Barak Y, Metzker A (1995). Pyodermagangrenosum and sterile multifocal osteomyelitis preceding the appearance of Takayasu arteritis. Pediatr Dermatol.

[14] Edwards TL, Stapleton FB, Bond MJ, Barrett FF (1986). Sweet’s syndrome with multifocal sterile osteomyelitis. Arch Pediatr Adolesc Med.

[15] Pelkonen P, Ryoppy S, Jaaskelainen J, Rapola J, Repo H, Kaitila I (1988). Chronic osteomyelitis-like disease with negative bacterial cultures. Arch Pediatr Adolesc Med..

[16] Rohekar G, Inman RD (2006). Conundrums in nosology: synovitis, acne, pustolosis, hyperostosisand osteitis syndrome and spondylarthritis. Ann Rheum.

[17] Hoffmann SR, Kubasch AS, Ioannidis C (2015). Alterated expression of IL-10 family citokynie in monocytes from CRMO patients result in enhanced IL-1 b expression and relase. Clin Immunol.

[18] Scianaro R, Insalaco A, Bracci Laudiero R (2014). Deregulation of the IL1beta axis in chronic recurrent multifocal osteomyelitis. Pediatr Rheumatol Online J.

[19] Wipff J, Adamsbaum C, Kahan A, Job-Deslandre C (2011). Chronic recurrent multifocal osteomyelitis. Bone Spine.

[20] Cox AJ, Zhao Y, Ferquson PJ (2017). Chronic Recurrent Multifocal Osteomyelitis and Related Diseases-Update on Pathogenesis. Curr Rheumatol Rep.

[21] Wipff J, Adamsbaum C, Kahan A (2011). Chronic recurrent multifocal osteomyelitis. Jt Bon Spine.

[22] Guerin-Pfyffer S, Guillaume-Czitrom S, Tammam S (2012). Evaluation of chronic recurrent multifocal osteitis in children by whole-body magnetic resonance imaging. Jt Bone Spine.

[23] Jansson A, Renner ED, Ramser J (2007). Classification of non-bacterial osteitis: retrospective study of clinical, immunological and genetic aspects in 89 patients. Rheumatology (Oxford).

[24] Harel L, Hashkes PJ, Lapidus S, et al. The First International Conference on Periodic Fever, Aphthous Stomatitis, Pharyngitis, Adenitis Syndrome J Pediatr. 2017;(17)31437–3. 10.1016/j.jpeds.2017.10.03. 10.1016/j.jpeds.2017.10.034 29246466

[25] Cox AJ, Zhao Y, Ferquson PJ Chronic Recurrent Multifocal Osteomyelitis and Related Diseases an Update on Pathogenesis CurrRheumatol Rep. 2017;19(4):18. 10.1007/s11926-017-0645-9. 10.1007/s11926-017-0645-9 PMC5545929 28361334

[26] T.Von Kalle, N.Heim, T.Hospach et al. Typical pattern of bone involvement in whole-body MRI of patients with chronic recurrent multifocal osteomyelitis (CRMO) Rofo 2013;185(7):655–61. 10.1055/s-0033-1335283 23696017

[27] Ramay R, Chuc C et al. Chronic recurrent multifocal osteomyelitis in Crohn's Disease, complete resolution with anti TNF-α therapy. J Pediatr Gastrointestinal Nutr. 2016. 10.1097/MPG.0000000000001228 27050047

[28] Ishikawa-Nakayama K, Sugiyama E, Sawazaki S, Taki H, Kobayashi M, Koizumi F (2000). Chronic recurrent multifocal osteomyelitis showing marked improvement with corticosteroid treatment. J Rheumatol.

[29] Kaiser D, Bolt I, Hofer M (2015). Pediatric chronic non bacterial osteomyelitis in children : a retrospective multicenter study. Pediatr Rheumatol online J.

[30] Taddio A, Zennaro F. Pastore S an update on the pathogenesis and treatment of Chronic recurrent multifocal osteomyelitis in children. Pediatr Drugs. 2017; 10.1007/s40272-017-0226-4. 10.1007/s40272-017-0226-4 28401420

[31] Roderick M, Shah R, Finn A, Rmanan AV. Efficacy of pamidronate therapy in children with chronic non-bacterial osteitis: disease activity assessment by whole body magnetic resonance imaging. Rheumatology (Oxford). 2014. 10.1093/rheumatology/keu226 24899664

[32] Zhao B, Grimes SN, Li S, Hu X, Ivashkiv LB (2012). TNF-induced osteoclastogenesis and inflammatory bone resorption are inhibited by transcription factor RBP-J. J Exp Med.

[33] Scianaro R, Insalaco A, Bracci LL (2014). Deregulation of the IL-1β axis in chronic recurrent multifocal osteomyelitis. Pediatr Rheumatol.

